# Transient Albuminuria as It Occurs, Particularly in Children and Adolescents, in Apparent Health

**Published:** 1882-02

**Authors:** 


### TRANSIENT ALBUMINURIA AS IT OCCURS, PARTICULARLY IN CHILDREN AND ADOLESCENTS, IN APPARENT HEALTH.

Dr. Frank P. Kinnicutt read a paper on the above subject, in which he first alluded to the practical importance of the occurrence of transient albuminuria, to which especial attention had been directed during the past few years by Sir William Gull, George Johnson, Saundby, Moxon, and others, and then gave a resume of the various theories which, from time to time, have been offered in explanation of this phenomenon.

That which the author of the paper suggested as the most probable was the following: The transient albuminuria in persons apparently healthy is due to slowing of the blood-current in the glomerular vessels, dependent upon temporary vaso-motor disturbances, with alteration, also temporary, in the glomerular epithelium. He farther believed that the real source of irritation was to be found in the temporary presence of imperfectly oxygenized matters in the renal circulation—in other words, transient oxaluria and lithuria.

Dr. Kinnicutt then reviewed the cases of transient albuminuria which have been reported by various observers. In 1873, Sir William Gull said that, in his experience, it occurred in young, growing men and boys almost as frequently as spermatorrhœa. In 1878, Moxon, in "Guy's Hospital Reports," reported nineteen cases. Dr. Clement Dukes, in the *British Medical Journal*, November, 1878, reported several cases, which, in many respects, differed from those given by Dr. Moxon; and in the same journal for November 10, 1881, Dukes had reversed his opinion, and stated that he regarded albuminuria as evidence of true Bright's disease. Dr. George Johnson also, in the *British Medical Journal,* had directed attention to the question of temporary albuminuria, and had expressed the opinion that the smallest trace was always pathological and never physiological. Reference was also made to cases reported by Saundby, of Manchester, and others.

Dr. Kinnicutt then gave a brief sketch of each of his own cases. The first three patients were young men, aged twenty-three, twenty-one, and seventeen respectively. In each case there was a large amount of albumen found in the urine, together with oxalate of lime and uric acid crystals. The chief subjective symptoms were: sense of weariness, lassitude, inaptitude for either mental or physical labor, headache slightly in the morning, impairment of appetite, etc. Exercise in the open air and mineral waters were prescribed, together with regulation of diet, occupation, and habits, and both the subjective and the urinary symptoms disappeared permanently. The histories of several other cases were given, occurring in patients whose ages varied from five to twenty-two.

His own observations seemed to show that temporary albuminuria, as it occurs in children and adolescents in apparent health, may be traced, in a large number of instances, to a transient oxaluria or lithuria, and he suggested that the sequence of events in the causation of the albuminuria is as follows:

*First.*—The temporary presence of a large amount of imperfectly oxygenated matter in the circulation.

*Second.*—Disturbances of the general nervous system, in which the vaso-motor system of the kidney shares, or one confined to the vaso-motor system of the kidney in its elimination of these products of a faulty digestion.

*Third.*—A transient dilation of blood-vessels in the kidney, and a retardation of the blood-current in the glomerular vessels, with possibly consequent alteration in the functions of the glomerular epithelium, also of a temporary nature.—*Med. Record.*

